# Supplementary material for: Optimal minimal residual disease threshold in pediatric acute myeloid leukemia: A retrospective cohort study based on the TARGET database
Source: PLoS Med. 2026 May 8;23(5):e1005088. doi: 10.1371/journal.pmed.1005088 (PMC13155632; doi:10.1371/journal.pmed.1005088)
Supplement: S1 Code — (ZIP) [file pmed.1005088.s002.zip › S2 code/PROJ8_14_tbl1/PROJ8_14_tbl1.htm]

## Kaplan Meier Survival Curve

Outcome: First Event
Time: EFS(years)
Landmark analysis for time segment: EFS(years) <= 5
Log rank test: implements the G-rho family of Harrington and Fleming (1982), with weights on each death of S(t)^rho, where S is the Kaplan-Meier estimate of survival. With rho = 0 this is the log-rank or Mantel-Haenszel test.

|  |  |  |  |
| --- | --- | --- | --- |
|  | N | Observed | Expected |
| factor(X23.CS)=0 | 72 | 25 | 49.4912 |
| factor(X23.CS)=1 | 56 | 48 | 23.5088 |

Chisq=
38.8788
on
1
degree of freedom, p=
<0.0001
Survival table

|  |  |  |  |  |  |  |  |
| --- | --- | --- | --- | --- | --- | --- | --- |
|  | EFS(years) | N.Risk | N.Event | N.Censor | Survival | 95%CI Low | 95%CI Upp |
| factor(X23.CS)=0 | 0.21 | 72 | 2 | 0 | 0.9722 | 0.9350 | 1.0000 |
| factor(X23.CS)=0 | 0.22 | 70 | 1 | 0 | 0.9583 | 0.9133 | 1.0000 |
| factor(X23.CS)=0 | 0.24 | 69 | 1 | 0 | 0.9444 | 0.8930 | 0.9989 |
| factor(X23.CS)=0 | 0.41 | 68 | 1 | 0 | 0.9306 | 0.8737 | 0.9912 |
| factor(X23.CS)=0 | 0.44 | 67 | 2 | 0 | 0.9028 | 0.8369 | 0.9739 |
| factor(X23.CS)=0 | 0.45 | 65 | 1 | 0 | 0.8889 | 0.8192 | 0.9645 |
| factor(X23.CS)=0 | 0.55 | 64 | 2 | 0 | 0.8611 | 0.7848 | 0.9448 |
| factor(X23.CS)=0 | 0.64 | 62 | 2 | 0 | 0.8333 | 0.7515 | 0.9240 |
| factor(X23.CS)=0 | 0.66 | 60 | 1 | 0 | 0.8194 | 0.7352 | 0.9133 |
| factor(X23.CS)=0 | 0.67 | 59 | 1 | 0 | 0.8056 | 0.7191 | 0.9024 |
| factor(X23.CS)=0 | 0.74 | 58 | 1 | 0 | 0.7917 | 0.7032 | 0.8913 |
| factor(X23.CS)=0 | 0.76 | 57 | 1 | 0 | 0.7778 | 0.6874 | 0.8800 |
| factor(X23.CS)=0 | 0.81 | 56 | 1 | 0 | 0.7639 | 0.6718 | 0.8686 |
| factor(X23.CS)=0 | 0.84 | 55 | 2 | 0 | 0.7361 | 0.6410 | 0.8453 |
| factor(X23.CS)=0 | 0.85 | 53 | 1 | 0 | 0.7222 | 0.6258 | 0.8335 |
| factor(X23.CS)=0 | 0.91 | 52 | 1 | 0 | 0.7083 | 0.6108 | 0.8215 |
| factor(X23.CS)=0 | 1.05 | 51 | 1 | 0 | 0.6944 | 0.5958 | 0.8094 |
| factor(X23.CS)=0 | 1.11 | 50 | 0 | 2 | 0.6944 | 0.5958 | 0.8094 |
| factor(X23.CS)=0 | 1.15 | 48 | 0 | 1 | 0.6944 | 0.5958 | 0.8094 |
| factor(X23.CS)=0 | 1.16 | 47 | 1 | 0 | 0.6797 | 0.5798 | 0.7967 |
| factor(X23.CS)=0 | 1.25 | 46 | 0 | 1 | 0.6797 | 0.5798 | 0.7967 |
| factor(X23.CS)=0 | 1.37 | 45 | 0 | 1 | 0.6797 | 0.5798 | 0.7967 |
| factor(X23.CS)=0 | 1.61 | 44 | 0 | 1 | 0.6797 | 0.5798 | 0.7967 |
| factor(X23.CS)=0 | 1.83 | 43 | 0 | 1 | 0.6797 | 0.5798 | 0.7967 |
| factor(X23.CS)=0 | 2.14 | 42 | 1 | 0 | 0.6635 | 0.5621 | 0.7831 |
| factor(X23.CS)=0 | 2.37 | 41 | 1 | 0 | 0.6473 | 0.5446 | 0.7693 |
| factor(X23.CS)=0 | 2.46 | 40 | 0 | 1 | 0.6473 | 0.5446 | 0.7693 |
| factor(X23.CS)=0 | 2.73 | 39 | 0 | 1 | 0.6473 | 0.5446 | 0.7693 |
| factor(X23.CS)=0 | 3.04 | 38 | 0 | 2 | 0.6473 | 0.5446 | 0.7693 |
| factor(X23.CS)=0 | 3.28 | 36 | 0 | 1 | 0.6473 | 0.5446 | 0.7693 |
| factor(X23.CS)=0 | 3.48 | 35 | 0 | 1 | 0.6473 | 0.5446 | 0.7693 |
| factor(X23.CS)=0 | 3.74 | 34 | 0 | 1 | 0.6473 | 0.5446 | 0.7693 |
| factor(X23.CS)=0 | 3.84 | 33 | 0 | 1 | 0.6473 | 0.5446 | 0.7693 |
| factor(X23.CS)=0 | 3.89 | 32 | 0 | 1 | 0.6473 | 0.5446 | 0.7693 |
| factor(X23.CS)=0 | 3.9 | 31 | 0 | 1 | 0.6473 | 0.5446 | 0.7693 |
| factor(X23.CS)=0 | 4.05 | 30 | 0 | 1 | 0.6473 | 0.5446 | 0.7693 |
| factor(X23.CS)=0 | 4.6 | 29 | 0 | 1 | 0.6473 | 0.5446 | 0.7693 |
| factor(X23.CS)=0 | 4.73 | 28 | 0 | 1 | 0.6473 | 0.5446 | 0.7693 |
| factor(X23.CS)=0 | 5 | 27 | 0 | 27 | 0.6473 | 0.5446 | 0.7693 |
| factor(X23.CS)=1 | 0.13 | 56 | 2 | 0 | 0.9643 | 0.9169 | 1.0000 |
| factor(X23.CS)=1 | 0.15 | 54 | 1 | 0 | 0.9464 | 0.8893 | 1.0000 |
| factor(X23.CS)=1 | 0.16 | 53 | 2 | 0 | 0.9107 | 0.8390 | 0.9885 |
| factor(X23.CS)=1 | 0.17 | 51 | 2 | 0 | 0.8750 | 0.7925 | 0.9661 |
| factor(X23.CS)=1 | 0.18 | 49 | 2 | 0 | 0.8393 | 0.7484 | 0.9412 |
| factor(X23.CS)=1 | 0.19 | 47 | 3 | 0 | 0.7857 | 0.6853 | 0.9009 |
| factor(X23.CS)=1 | 0.2 | 44 | 1 | 0 | 0.7679 | 0.6649 | 0.8868 |
| factor(X23.CS)=1 | 0.21 | 43 | 1 | 0 | 0.7500 | 0.6447 | 0.8724 |
| factor(X23.CS)=1 | 0.22 | 42 | 3 | 0 | 0.6964 | 0.5858 | 0.8279 |
| factor(X23.CS)=1 | 0.25 | 39 | 1 | 0 | 0.6786 | 0.5666 | 0.8126 |
| factor(X23.CS)=1 | 0.28 | 38 | 2 | 0 | 0.6429 | 0.5289 | 0.7814 |
| factor(X23.CS)=1 | 0.29 | 36 | 1 | 0 | 0.6250 | 0.5102 | 0.7656 |
| factor(X23.CS)=1 | 0.32 | 35 | 1 | 0 | 0.6071 | 0.4918 | 0.7495 |
| factor(X23.CS)=1 | 0.33 | 34 | 1 | 0 | 0.5893 | 0.4735 | 0.7333 |
| factor(X23.CS)=1 | 0.34 | 33 | 1 | 0 | 0.5714 | 0.4555 | 0.7169 |
| factor(X23.CS)=1 | 0.42 | 32 | 1 | 0 | 0.5536 | 0.4375 | 0.7004 |
| factor(X23.CS)=1 | 0.49 | 31 | 1 | 0 | 0.5357 | 0.4198 | 0.6836 |
| factor(X23.CS)=1 | 0.5 | 30 | 1 | 0 | 0.5179 | 0.4022 | 0.6668 |
| factor(X23.CS)=1 | 0.53 | 29 | 1 | 0 | 0.5000 | 0.3848 | 0.6497 |
| factor(X23.CS)=1 | 0.62 | 28 | 1 | 0 | 0.4821 | 0.3675 | 0.6325 |
| factor(X23.CS)=1 | 0.72 | 27 | 1 | 0 | 0.4643 | 0.3504 | 0.6151 |
| factor(X23.CS)=1 | 0.84 | 26 | 3 | 0 | 0.4107 | 0.3001 | 0.5621 |
| factor(X23.CS)=1 | 0.91 | 23 | 3 | 0 | 0.3571 | 0.2513 | 0.5075 |
| factor(X23.CS)=1 | 1.07 | 20 | 1 | 0 | 0.3393 | 0.2354 | 0.4890 |
| factor(X23.CS)=1 | 1.09 | 19 | 1 | 0 | 0.3214 | 0.2197 | 0.4703 |
| factor(X23.CS)=1 | 1.15 | 18 | 2 | 0 | 0.2857 | 0.1888 | 0.4323 |
| factor(X23.CS)=1 | 1.17 | 16 | 1 | 0 | 0.2679 | 0.1737 | 0.4130 |
| factor(X23.CS)=1 | 1.18 | 15 | 1 | 0 | 0.2500 | 0.1588 | 0.3935 |
| factor(X23.CS)=1 | 1.39 | 14 | 2 | 1 | 0.2143 | 0.1298 | 0.3538 |
| factor(X23.CS)=1 | 1.45 | 11 | 1 | 0 | 0.1948 | 0.1141 | 0.3327 |
| factor(X23.CS)=1 | 1.55 | 10 | 2 | 0 | 0.1558 | 0.0840 | 0.2893 |
| factor(X23.CS)=1 | 1.74 | 8 | 1 | 0 | 0.1364 | 0.0697 | 0.2669 |
| factor(X23.CS)=1 | 3.87 | 7 | 0 | 1 | 0.1364 | 0.0697 | 0.2669 |
| factor(X23.CS)=1 | 4.33 | 6 | 0 | 1 | 0.1364 | 0.0697 | 0.2669 |
| factor(X23.CS)=1 | 5 | 5 | 0 | 5 | 0.1364 | 0.0697 | 0.2669 |

|  |  |  |  |  |  |  |  |  |  |
| --- | --- | --- | --- | --- | --- | --- | --- | --- | --- |
| X23.CS | records | n.max | n.start | events | \*rmean | \*se(rmean) | median | 0.95LCL | 0.95UCL |
| factor(X23.CS)=0 | 72 | 72 | 72 | 25 | 3.509 | 0.242 | NA | NA | NA |
| factor(X23.CS)=1 | 56 | 56 | 56 | 48 | 1.22 | 0.212 | 0.575 | 0.32 | 1.07 |

Landmark analysis for time segment: EFS(years) > 5
Log rank test: implements the G-rho family of Harrington and Fleming (1982), with weights on each death of S(t)^rho, where S is the Kaplan-Meier estimate of survival. With rho = 0 this is the log-rank or Mantel-Haenszel test.

|  |  |  |  |
| --- | --- | --- | --- |
|  | N | Observed | Expected |
| factor(X23.CS)=0 | 27 | 3 | 3.7667 |
| factor(X23.CS)=1 | 5 | 1 | 0.2333 |

Chisq=
2.9227
on
1
degree of freedom, p=
0.0873
Survival table

|  |  |  |  |  |  |  |  |
| --- | --- | --- | --- | --- | --- | --- | --- |
|  | EFS(years) | N.Risk | N.Event | N.Censor | Survival | 95%CI Low | 95%CI Upp |
| factor(X23.CS)=0 | 5.05 | 27 | 0 | 1 | 1.0000 | 1.0000 | 1.0000 |
| factor(X23.CS)=0 | 5.14 | 26 | 0 | 1 | 1.0000 | 1.0000 | 1.0000 |
| factor(X23.CS)=0 | 5.32 | 25 | 0 | 1 | 1.0000 | 1.0000 | 1.0000 |
| factor(X23.CS)=0 | 5.35 | 24 | 0 | 1 | 1.0000 | 1.0000 | 1.0000 |
| factor(X23.CS)=0 | 5.7 | 23 | 0 | 1 | 1.0000 | 1.0000 | 1.0000 |
| factor(X23.CS)=0 | 5.74 | 22 | 0 | 1 | 1.0000 | 1.0000 | 1.0000 |
| factor(X23.CS)=0 | 5.8 | 21 | 0 | 1 | 1.0000 | 1.0000 | 1.0000 |
| factor(X23.CS)=0 | 6 | 20 | 0 | 1 | 1.0000 | 1.0000 | 1.0000 |
| factor(X23.CS)=0 | 6.15 | 19 | 0 | 1 | 1.0000 | 1.0000 | 1.0000 |
| factor(X23.CS)=0 | 6.4 | 18 | 0 | 1 | 1.0000 | 1.0000 | 1.0000 |
| factor(X23.CS)=0 | 6.45 | 17 | 3 | 0 | 0.8235 | 0.6609 | 1.0000 |
| factor(X23.CS)=0 | 6.59 | 14 | 0 | 1 | 0.8235 | 0.6609 | 1.0000 |
| factor(X23.CS)=0 | 6.65 | 13 | 0 | 2 | 0.8235 | 0.6609 | 1.0000 |
| factor(X23.CS)=0 | 6.75 | 11 | 0 | 1 | 0.8235 | 0.6609 | 1.0000 |
| factor(X23.CS)=0 | 6.79 | 10 | 0 | 1 | 0.8235 | 0.6609 | 1.0000 |
| factor(X23.CS)=0 | 6.89 | 9 | 0 | 1 | 0.8235 | 0.6609 | 1.0000 |
| factor(X23.CS)=0 | 6.92 | 8 | 0 | 1 | 0.8235 | 0.6609 | 1.0000 |
| factor(X23.CS)=0 | 6.94 | 7 | 0 | 1 | 0.8235 | 0.6609 | 1.0000 |
| factor(X23.CS)=0 | 7 | 6 | 0 | 2 | 0.8235 | 0.6609 | 1.0000 |
| factor(X23.CS)=0 | 7.36 | 4 | 0 | 1 | 0.8235 | 0.6609 | 1.0000 |
| factor(X23.CS)=0 | 7.53 | 3 | 0 | 1 | 0.8235 | 0.6609 | 1.0000 |
| factor(X23.CS)=0 | 8.15 | 2 | 0 | 1 | 0.8235 | 0.6609 | 1.0000 |
| factor(X23.CS)=0 | 8.28 | 1 | 0 | 1 | 0.8235 | 0.6609 | 1.0000 |
| factor(X23.CS)=1 | 5.55 | 5 | 0 | 1 | 1.0000 | 1.0000 | 1.0000 |
| factor(X23.CS)=1 | 6.04 | 4 | 0 | 1 | 1.0000 | 1.0000 | 1.0000 |
| factor(X23.CS)=1 | 6.19 | 3 | 0 | 1 | 1.0000 | 1.0000 | 1.0000 |
| factor(X23.CS)=1 | 6.39 | 2 | 0 | 1 | 1.0000 | 1.0000 | 1.0000 |
| factor(X23.CS)=1 | 6.56 | 1 | 1 | 0 | 0.0000 |  |  |

|  |  |  |  |  |  |  |  |  |  |
| --- | --- | --- | --- | --- | --- | --- | --- | --- | --- |
| X23.CS | records | n.max | n.start | events | \*rmean | \*se(rmean) | median | 0.95LCL | 0.95UCL |
| factor(X23.CS)=0 | 27 | 27 | 27 | 3 | 7.249 | 0.09 | NA | NA | NA |
| factor(X23.CS)=1 | 5 | 5 | 5 | 1 | 6.56 | 0 | 6.56 | NA | NA |

Use subset of data: (!is.na(X5) & (X5==3))
Created by EmpowerStats (www.empowerstats.com) and R on 2025-10-07
